# Combined Use of Astragalus Polysaccharide and Berberine Attenuates Insulin Resistance in IR-HepG2 Cells *via* Regulation of the Gluconeogenesis Signaling Pathway

**DOI:** 10.3389/fphar.2019.01508

**Published:** 2019-12-23

**Authors:** Zhu-Jun Mao, Min Lin, Xin Zhang, Lu-Ping Qin

**Affiliations:** ^1^ College of Pharmaceutical Sciences, Zhejiang Chinese Medical University, Hangzhou, China; ^2^ College of Basic Medical Sciences, Zhejiang Chinese Medical University, Hangzhou, China

**Keywords:** astragalus polysaccharide, berberine, insulin resistance, IR-HepG2 cell model, intracellular calcium flux, gluconeogenesis signaling pathway

## Abstract

Insulin resistance (IR) is likely to induce metabolic syndrome and type 2 diabetes mellitus (T2DM). Gluconeogenesis (GNG) is a complex metabolic process that may result in glucose generation from certain non-carbohydrate substrates. Chinese herbal medicine astragalus polysaccharides and berberine have been documented to ameliorate IR, and combined use of astragalus polysaccharide (AP) and berberine (BBR) are reported to synergistically produce an even better effect. However, what change may occur in the GNG signaling pathway of IR-HepG2 cells in this synergistic effect and whether AP-BBR attenuates IR by regulating the GNG signaling pathway remain unclear. For the first time, we discovered in this study that the optimal time of IR-HepG2 cell model formation was 48 h after insulin intervention. AP-BBR attenuated IR in HepG2 cells and the optimal concentration was 10 mg. AP-BBR reduced the intracellular H_2_O_2_ content with no significant effect on apoptosis of IR-HepG2 cells. In addition, a rapid change was observed in intracellular calcium current of the IR-HepG2 cell model, and AP-BBR intervention attenuated this change markedly. The gene sequencing results showed that the GNG signaling pathway was one of the signaling pathways of AP-BBR to attenuate IR in IR-Hepg2 cells. The expression of p-FoxO1^Ser256^ and PEPCK protein was increased, and the expression of GLUT2 protein was decreased significantly in the IR-HepG2 cell model, and both of these effects could be reversed by AP-BBR intervention. AP-BBR attenuated IR in IR-HepG2 cells, probably by regulating the GNG signaling Pathway.

## Introduction

Diabetes mellitus is classified as type 1 (T1DM) and type 2 (T2DM). Clinically, more than 90% DM patients belong to T2DM. Insulin resistance (IR) is a pathological condition in which a target cell or a target organ fails to respond normally to insulin, so that insulin is unable to stimulate glucose disposal, which is a serious risk factor leading to the onset of T2DM. Other than T2DM, IR is forerunner for the development of many metabolic diseases, such as hypertension, obesity, and atherosclerosis. Therefore, ameliorating the IR status is an effective approach for the treatment of T2DM (Rodriguez-Gutierrez et al., 2018). IR is a state in which the response of target organs to insulin is decreased, or the normal dose of insulin secreted by pancreatic islet β cells produces less than normal biological effects. The IR theory has been generally accepted as the theoretical foundation for the study of T2DM by the medical community.

Our previous study (Zhu-jun et al., 2017)showed that combined use of astragalus polysaccharide (AP) and berberine (BBR) could synergistically produce a better therapeutic effect than either of them alone by promoting the basic secretion ability of IR-INS-1 cells, and this research achievement is on the way of applying for a patent in China (patent application no.: CN201810266347.9; publication/announcement no.: CN108478591A).

The liver is the main organ of glucose and lipid metabolism and plays an important role in energy metabolism of various tissues and organs including muscles and adipose tissues through regulation of insulin, glucagon, epinephrine, growth hormone, and other hormones (von Horn and Minor, 2019). The construction of an IR cell model is an important way to explore the pathological mechanism and drug development of IR-related diseases.

HepG2 cells originating from human hepatic embryonal tumor cells are a phenotype very similar to hepatocytes. It was reported that the number of insulin receptors on the surface of HepG2 cells decreased by 58% under the high-level insulin condition, and the remaining receptors then exhibited a 50% decrease in insulin internalization and degradation on a per receptor basis (Williams and Olefsky, 1990). The degree of such a decrease was positively correlated with the insulin level and the duration of stimulation, indicating that HepG2 is a good cell model to study IR pathogenesis and the mechanism of hypoglycemic drugs *in vitro*.

The results of gene sequencing in a previous study (Tsujimoto et al., 2015) showed that there were significant differences in glycolysis/gluconeogenesis pathway between the control and model groups, and between the model and drug administration groups. Gluconeogenesis (GNG) is a metabolic process of converting non-carbohydrates, such as amino acids, pyruvate, and glycerin, into glycogen or glucose, which is important for maintaining blood glucose levels within the physiological range.

Under normal physiological conditions, the liver is the main organ of GNG. It has rapid adaptation to metabolic shift between fed and fasting states through a reciprocal mechanism (Wahren and Ekberg, 2007): Hepatic gluconeogenic activity is suppressed in response to postprandial insulin secretion so as to limit glucose production. Conversely, hepatic GNG is stimulated in response to reduced insulin action and elevates glucagon secretion during fasting. The renal glycogenic ability is only 1/10 of the liver (Edgerton et al., 2009).GNG change is not obvious under normal dietary and metabolic conditions, but can undergo significant change in prolonged fasting or exercise and under low-carbohydrate diet conditions, or when there is a significant decrease in glycogen storage in the liver. GNG is an energy-consuming process that starts with two molecules of pyruvate and ends with the synthesis of one molecule of glucose, which requires six molecules of ATP/GTP. It produces a net 2ATP compared to glycolysis. In T2DM patients, the main source of endogenous glucose production is from GNG rather from glycogende composition. Increased hepatic GNG is a hallmark of T2DM, so maintaining the normal rate of GNG is the key to avoiding T2DM (Matsumoto et al., 2007).

However, what change may occur in the GNG signaling pathway of IR-HepG2 cells, and whether AP-BBR can attenuate IR by regulating the GNG signaling pathway remain unclear.

In the present study, we established a model of IR- HepG2 cells by insulin induction *in vitro* to determine the concentration and timing of stable insulin induction, and detect glucose uptake, reactive oxygen species (ROS) and apoptosis, CCK8 cell viability, the H_2_O_2_ concentration, and intracellular calcium ion in HepG2 cells to see whether AP-BBR attenuated IR by regulating the GNG signaling pathway.

## Materials and Methods

### Determination of Glucose Content in HepG2 Cells

#### HepG2 Cell Culture

HepG2 cells (Cell Bank of the Chinese Academy of Sciences, Shanghai, China) were cultured in 1640 medium (Hyclone, Beijing, China) containing 10% fetal bovine serum (FBS, Hyclone, Beijing, China) and 1× streptomycin in a 37°C 5% CO_2_ saturated humidity incubator. The normally cultured HepG2 cell lines in log phase were centrifuged at 100 grpm for 5 min, and 20000 cells/well were placed in a 96-well plate and incubated at 37°C.

#### Grouping and Drug Administration

The experiment was performed in 24-h control group, 24-h model group, 36-h control group, 36-h model group, 48-h control group, 48-h model group, 72-h control group and 72-h model group.

Insulin (Gibco, NY, USA) was diluted to a final concentration of 10^-6^ mol/L in complete medium. 200-µl insulin preparation was added into each well for the model group and an equal amount of complete medium was added into each well for the control group. Culture was performed in a 37°C 5% CO_2_ and saturated humidity incubator. The supernatant of the corresponding medium was collected according to the time point by centrifugation at 3000 r/min for 5 min and stored at −80°C for use.

#### Determination of the Glucose Content

The reagent for determining the glucose content (RSBIO, Shanghai, China) was balanced and configured at room temperature. 20-ml R1 reagent and 20-ml R2 reagent were mixed well. The EP tubes were marked as a blank tube, a calibration tube, a quality control tube, and a sample tube accordingly. 1000 µl working fluid was added to each tube; 10 µl distilled water was added to the blank tube; 10 µl calibration product was added to each tube; 10 µl quality control product was added to each tube of quality control tube; and 10 µl sample was added to each sample tube. Six samples were added in each group. After full mixing, the EP tubes were placed in 37°C water bath for 15 min. The 200-µl sample from each tube was transferred to a 96-well plate, and the absorbance was measured at 505 nm.

### Detection of HepG2 Cell Viability by CCK8

HepG2 cell culture was conducted in the following groups: a normal control group; an insulin model group; a 1-mg AP-BBR group; a 5-mg AP-BBR group, a 10-mg AP-BBR group; a 20-mg AP-BBR group; and a 40-mg AP-BBR group. Astragalus polysaccharide (AP) used in this study was provided by Shanghai Yuanye Biotechnology Co., Ltd (Shanghai, China; Lot No. B20562; AP is a kind of macromolecular active substance extracted from the dried roots of the leguminous plant Astragalus membranaceus or Astragalus membranaceus. It is mainly composed of 75.19% glucose and a small amount of fructose, galactose, arabinose, and xylose. AP is a neutral polysaccharide that makes the iodine liquid blue and has a melting point above 200°C.), and BBR was provided by Beijing Century Aoko Biotechnology Co., Ltd (Beijing, China; Lot No. BWB50136; Molecular formula: C20H18NO4; Molecular weight: 336.37; Melting point: 145°C; boiling point: 354.2°C at 760 mm Hg). AP-BBR was administered at a 1:1 mass ratio of AP: BBR. The above ratio was set up according to our previous studies on AP and BBR (Zitai et al., 2018; Zhu-jun et al., 2018). After successful establishment of the IR model, different concentrations of AP-BBR were added. The system of each pore was 200 µl. The experiment was repeated three times independently. After different time points of drug action, 10 μl CCK8 detection solution (UNOCI, Hangzhou, China) was added to each pore, and the reaction time was 2 h at 37°C. A blank control group was set up and treated in the same way without anything added. Optical density (OD) was measured at 450 and 650 nm. The results were calculated using the following equation: Cell survival rate (%) = OD value of experimental group/OD value of non-drug group × 100%.

### Detection of Apoptosis and ROS of HepG2 Cells by Flow Cytometry

#### Grouping

HepG2 cells were divided into three groups: a normal control group, a model group, and a 10-mg AP-BBR group. The original culture medium was retained in the wells to which 100 µl of the respective insulin concentration was added and treated for 48 h.

#### ROS Probe Incubation

The cry preserved tube containing 1.5-ml cells was taken out from the liquid nitrogen tank and quickly placed in water bath at 37°C for about 2 min. The cell suspension in the tube was transferred into a 15-ml centrifuge tube, to which 5 ml complete medium was added, and centrifuged at 300×g for 5 min at room temperature. After removing the supernatant, cells were re-suspended with a moderate amount of complete medium heavy precipitation, inoculated in a 10-cm petri dish, added with complete medium to 10 ml, and cultured again in 37°C and 5% CO_2,_ saturated humidity. 1 μl ROS probe (Beyotime, Shanghai, China) was added to the resuscitated cells in the proportion of 1:1000, mixed, incubated at 37°C for 20 min, and oscillated several times per 5 min. After 5 centrifugations, cell precipitation was collected, 1 ml PBS was resuscitated, and centrifuged at 500 × g for 5 min. 1 ml PBS was re-suspended. A negative control group was set up and treated in the same way without adding the probe.

#### Apoptosis Probe Incubation

The prepared cells were precipitated; 0.1 ml banding buffer was resuscitated; FITC probe 5 μl and PI probe 10 μl were added, mixed, incubated at room temperature for 5 min, and finally tested after addition of the banding buffer to 0.5 ml. The negative control group was treated in the same way without anything added. The single staining group was treated in the same way with addition of the FITC probe or PI probe only.

#### Detection of ROS and the Apoptosis Rate by Flow Cytometry

The ROS signal was detected by flow cytometry (BIO-RAD, USA) and FL-1A channel. The proportion of ROS-positive cells and the mean fluorescence intensity of cells were recorded. FITC signal was detected by flow cytometry and FL-1A channel, PI signal was detected by FL-2A channel, and the apoptosis rate was recorded.

### Determination of the Intracellular H_2_O_2_ Concentration in HepG2 Cells

Cells in each group were cultured according to “Methods 1.1”. The Krebs-Ringer buffer was prepared with double steamed water, and the pH was 7.5. The final concentration of Amplex Red was 100 mg, and the final concentration of HRP was 0.25 U/ml with 1× buffer. H_2_O_2_ standard was diluted by gradient, and the standard curve was drawn. After removing the original liquid from the 96-well plate and washing PBS, 50-μl working fluid was added to each cell and incubated away from light at 37°C for 30 min. The fluorescence intensity was measured at the excitation wavelength of 530 nm and the emission wavelength of 590 nm.

### Detection of Intracellular Ca^2+^ in HepG2 Cells by Laser Confocal Technique

#### Hepg2 Cells Were Cultured and an IR-HepG2 Model Was Established

Fluo-4 probe loading was performed as follows: the prepared cell confocal dish was washed with 1 ml PBS buffer without Ca ^2^^+^ and Mg ^2^^+^; after addition of the Fluo-4 probe at 1:1 000 and thorough mixing, cells were incubated at 37°C for 30 min, and then washed with PBS without Ca ^2^^+^ and Mg ^2^^+^ 3 times before testing.

#### Administration and Detection of Intracellular Ca^2+^ by Laser Confocal Microscopy

The working module of living cells was opened in advance, and the conditions were set as follows: 37°C, 5% CO _2_, saturated humidity. The incubated cell confocal dish samples were placed in the workbench, the control samples and the model samples were put immediately under 100 times the laser confocal microscope x 100 (OLYMPUS FV1000, Japan). The blue light channel (excitation wavelength 488 nm and emission wavelength 526 nm) was scanned by laser every 5 s, and the fluorescence intensity was recorded. The control group and model samples were taken from the blue light channel. After dripping AP-BBR with the final concentration of 10 mg, the fluorescence intensity was recorded, and the control group and the model sample were taken from the blue light channel (488 nm excitation wavelength and 526 nm emission wavelength). Immediately, the blue light channel (488 nm excitation wavelength and 526 nm emission wavelength) was scanned by laser every 5 s, and the fluorescence intensity was recorded. Cells were photographed continuously for 0 to 1,000 s.

### Transcriptome Sequencing and Pathway Enrichment Analysis

RNA isolation, purification, and quantification: Total RNA was isolated and purified using TRIzol reagent (Invitrogen, Carlsbad, CA, USA) following the manufacturer's procedure. The RNA amount and purity of each sample were quantified using NanoDrop ND-1000 (NanoDrop, Wilmington, DE, USA). The RNA integrity was assessed by Agilent 2100 with RIN number >7.0.

cDNA Library Construction: Poly(A) RNA was purified from total RNA (5 µg) using poly-T oligo-attached magnetic beads using two rounds of purification. Then, the poly(A) RNA was fragmented into small pieces using divalent cations under high temperature. Then the cleaved RNA fragments were reverse-transcribed to create the cDNA, which was subsequently used to synthesize U-labeled second-stranded DNAs with *Escherichia coli* DNA polymerase I, RNase H, and dUTP. An A-base was then added to the blunt ends of each strand, which were prepared for ligation to the indexed adapters. Each adapter contained a T-base overhang for ligating the adapter to the A-tailed fragmented DNA. Single- or dual-index adapters were ligated to the fragments, and size selection was performed with AMPureXP beads. After the heat-labile UDG enzyme treatment of the U-labeled second-stranded DNAs, the ligated products were amplified by PCR under the following conditions: initial denaturation at 95°C for 3 min, 8 cycles of denaturation at 98°C for 15 s, annealing at 60°C for 15 s, extension at 72°C for 30 s, and then final extension at 72°C for 5 min. The mean insert size for the final cDNA library was 300 bp (±50 bp). Finally, 150-bp paired-end sequencing was performed on an Illumina Hiseq 4000 (LC Bio, China) following the vendor's recommended protocol.

Pathway enrichment analysis: Using the DAVID database and mouse genome as background control, the differentially expressed genes were analyzed by gene ontology under “FunctionalAnnotation Chart” functional module. The differentially expressed genes were divided into three categories according to their functions: the biological process, the cell component, and the molecular function. Pathway analysis was carried out by KEGG analysis function.

### Analysis of Protein Expression

HepG2 cells were lysed in NP 40 lysis buffer and centrifuged at 12,000×*g* for 20 min at 4°C. The protein concentration was quantitated, and the protein lysate (20 μg proteins) was subjected to SDS-PAGE on 10% polyacrylamide gels and then electrotransferred onto PVDF membranes (Millipore, Billerica, MA, USA). The membranes were then blocked by 5% fat-free milk, and incubated respectively with FoxO1 antibody (78 kDa; 1:1000), Phospho-FoxO1 (Ser256) antibody (78 kDa; 1:1000), PEPCK (PCK2) antibody (71 kDa; 1:1000), GLUT2 antibody (53 kDa; 1:1000), and anti-GAPDH (36 kDa; 1:2000) (CST, Cambridge, MA, U.S.) at 4°C overnight. Horseradish peroxidase (HRP)-conjugated secondary antibodies (rabbit or mouse) were used to detect bound antibodies, which were visualized by enhanced chemiluminescence (ECL, Millipore, Billerica, MA, USA) on a VersaDoc 4000 MP (BIO-RAD, USA) workstation. Using the ImageJ software, densitometric analysis was performed to determine the relative expression level of the target protein, which was standardized to GAPDH (Bethesda, Maryland. USA).

### Statistical Analysis

Data concerning miRNA expression were analyzed with independent sample t-test. All the other data were analyzed with one-way ANOVA followed by LSD when equal variances assumed, and Dunnett's-T3 when equal variances not assumed. The results are expressed as the means ± SEM. All statistical analyses were performed by using SPSS23. *p* < 0.05 was considered statistically significant. Figures were drawn by GraphPad Prism 6.02 and Adobe Illustrator CS6.

## Results

### Determination of Glucose Content in HepG2 Cells

**Figure1:**
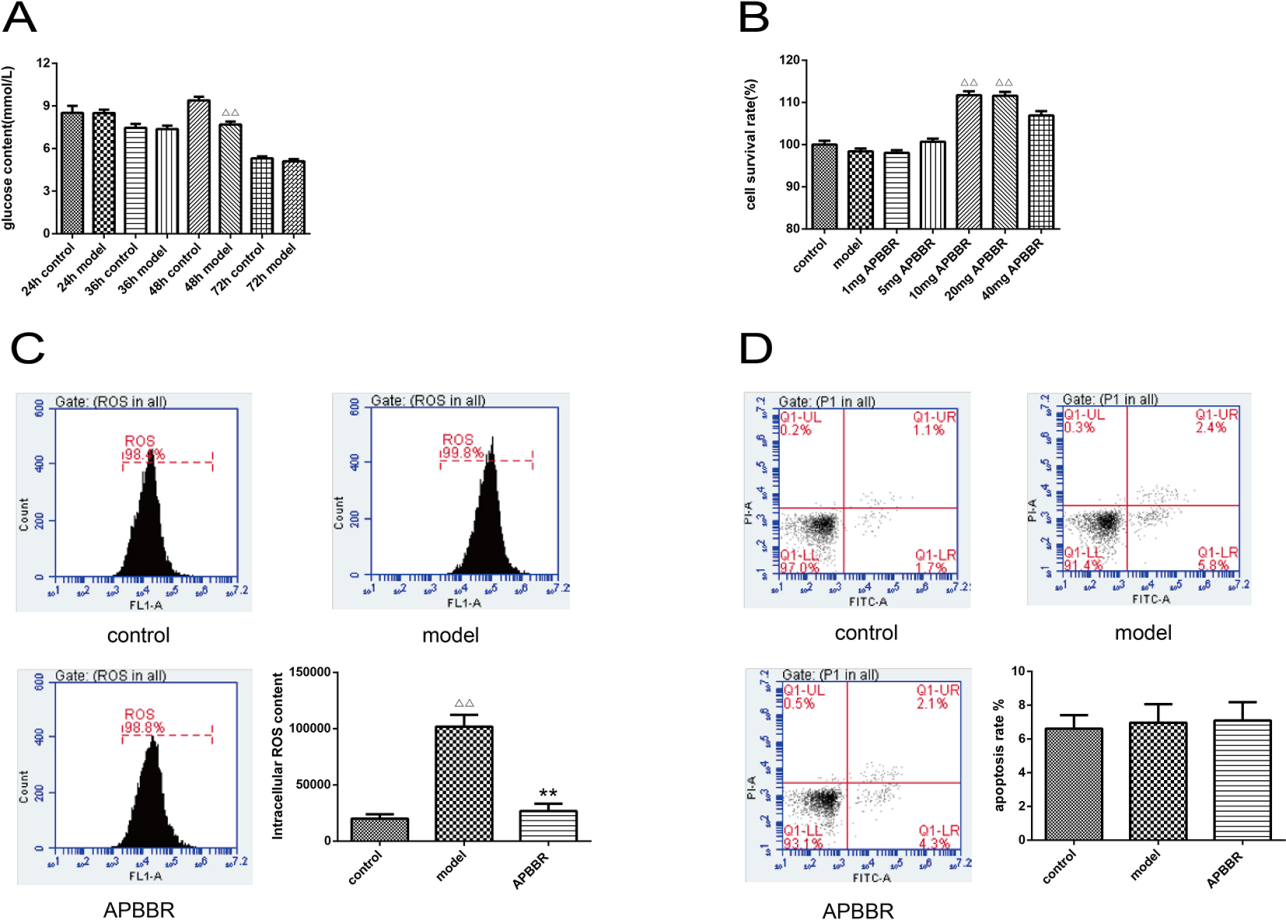
The glucose concentration of HepG2 cells **(A)**. Data are shown by mean ± SEM (n = 3), 48 h control group *vs.* 48 h model group, ^ΔΔ^ *P* < 0.01. Proliferation activity of HepG2 cells **(B)**, AP-BBR group *vs*. model group, **P < 0.01. The ROS content in HepG2 cells **(C)**, model group *vs*. control group, ^ΔΔ^ *P* < 0.01, AP-BBR group *vs*. model group, **P < 0.01. Apoptosis rate of HepG2 cells **(D)**, model group *vs*. control group, *P* > 0.05, AP-BBR group *vs*. model group, *P* > 0.05.

The results of intracellular glucose content determination in HepG2 cells are shown in **Figure 1A**. The best time for the HepG2 insulin model was at 48 h of insulin intervention.

### Result of the Cell Survival Rate of HepG2 Cells

CCK8 assay was performed to detect the cell survival rate of HepG2 cells. As shown in **Figure 1B**, the survival rate of IR-HepG2 cells was increased after AP-BBR administration. The optimal dosage of AP-BBR was 10 and 20 mg. For convenience, 10 mg was used in the subsequent experiments.

### Determination of ROS Content in HepG2 Cells

ROS content in HepG2 cells was detected by flow cytometry (**Figure 1C**). The results showed that the level of ROS in IR-HepG2 cells increased significantly, and the level of ROS decreased significantly in AP-BBR group. The results showed that the level of ROS in IR-HepG2 cells increased significantly, and AP-BBR decreased the level of ROS in the IR-HepG2 cell model.

### The Apoptosis Rate of HepG2 Cells

The effect of AP-BBR on apoptosis of IR-HepG2 cells was detected by flow cytometry, and the results are shown in **Figure 1D**. The results showed that the IR model and AP-BBR treatment had no significant effect on apoptosis of HepG2 cells.

### H_2_O_2_ Concentration in HepG2 Cells

**Figure 2:**
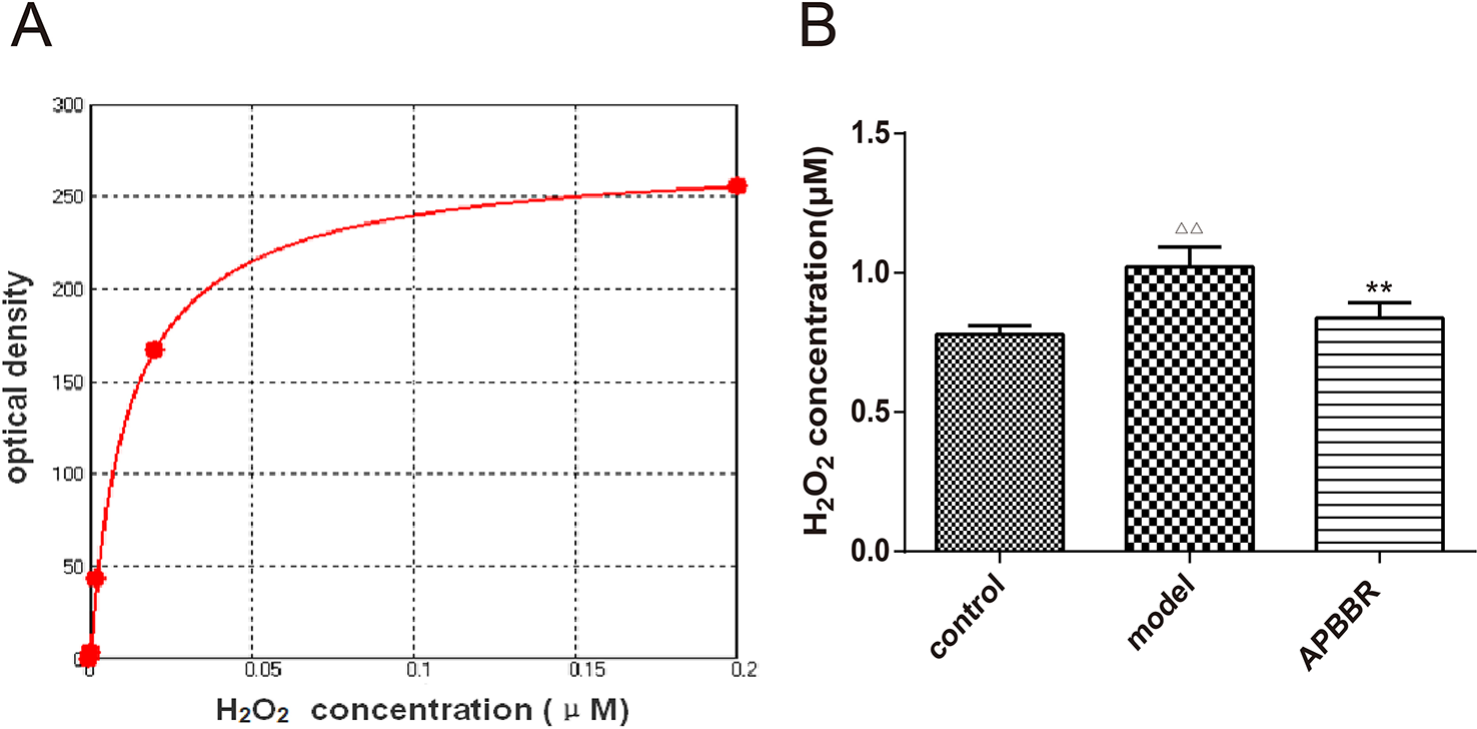
H_2_O_2_ standard curve **(A)**. X axis: concentration (mM); Y axis: fluorescence OD value. Four-parameter Logistic curve fitting, equation: y = (A -D)/[1 + (x/C)^B] + D.A = 274.48194; B = -0.92136; C = 0.01225; D = -1.30186; r^2 = 0.99990. H_2_O_2_ concentration in HepG2 cells **(B)**. Data are shown by mean ± SEM (n = 3). ^ΔΔ^ *P* < 0.01 showing a significant difference as compared with control group; **P < 0.01 showing a critical difference as compared with model group (n = 3).

The effect of AP-BBR on the concentration of H_2_O_2_ in IR-HepG2 cells was detected. As shown in **Figure 2**, the H_2_O_2_ content of IR-HepG2 cells was significantly increased, and the H_2_O_2_ content of IR-HepG2 cells was significantly decreased in AP-BBR group.

### Change in Intracellular Ca^2+^ in HepG2 Cells

**Figure 3:**
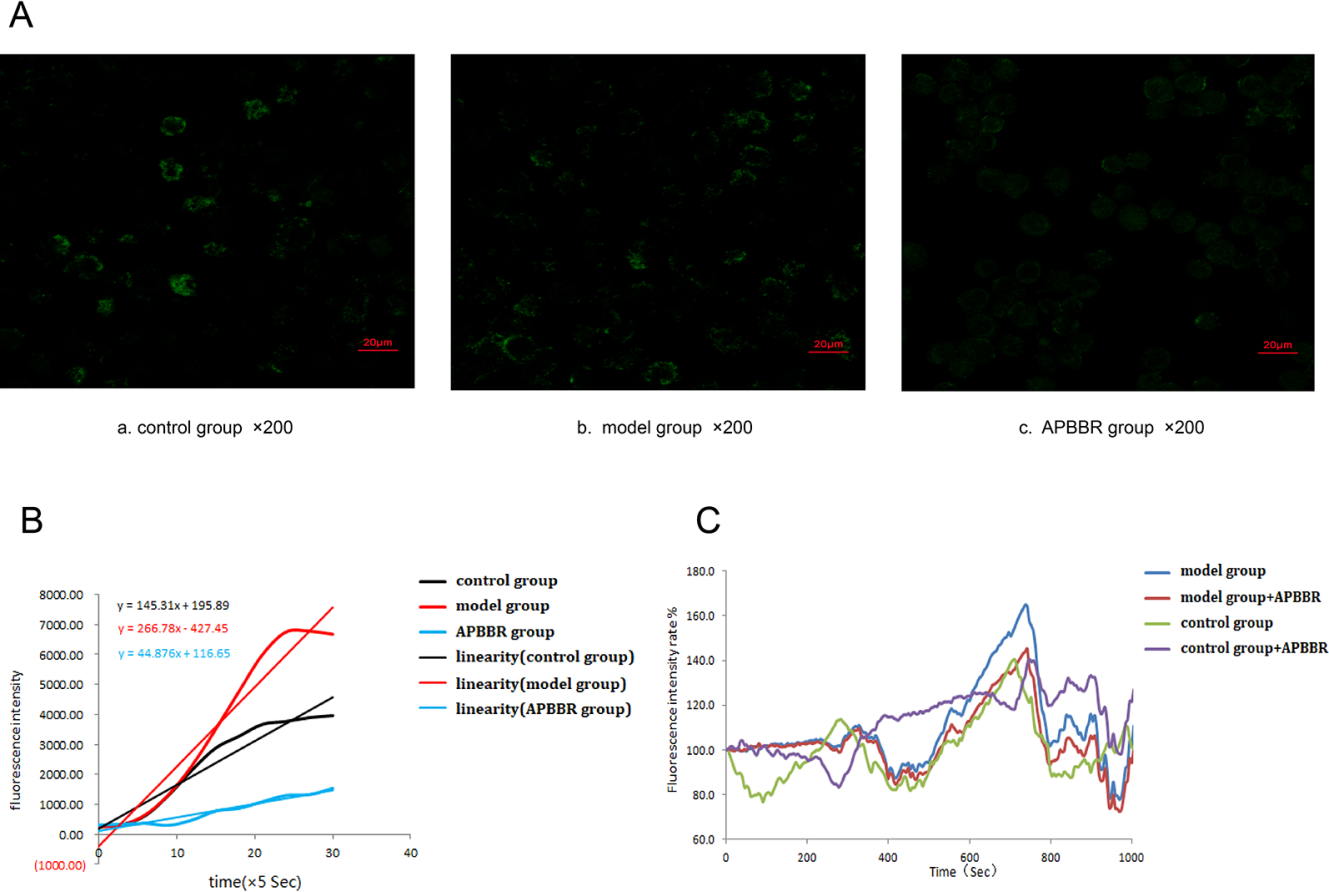
The photos of each HepG2 cells group under laser confocal microscope (×400) **(A)**. Calculation of slope by curve fitting of intracellular Ca^2+^ fluorescence intensity dynamics (0–150 s) **(B)**. Changes of intracellular Ca^2+^ fluorescence intensity in HepG2 cells(0–1000 s) **(C)**.

The photos of each HepG2 cell group under the laser confocal microscope are shown in **Figure 3A**.

The slope of intracellular Ca^2+^ fluorescence intensity dynamic (0–150 s) curve fitting is shown in **Figure 3B**. The results suggest that change in intracellular calcium flow became stronger and faster after modeling, and slower and weaker after AP-BBR administration.

The experimental results of laser confocal detection of intracellular Ca^2+^ change in HepG2 cells are shown in **Figure 3C** (the raw data is shown in **Supplementary Table 1**). These results suggest that the intracellular calcium flow changed rapidly in IR-HepG2 cells, and the intracellular calcium flow change was decreased after 10 mg AP-BBR administration.

### Results of Pathway Enrichment Analysis

**Figure 4:**
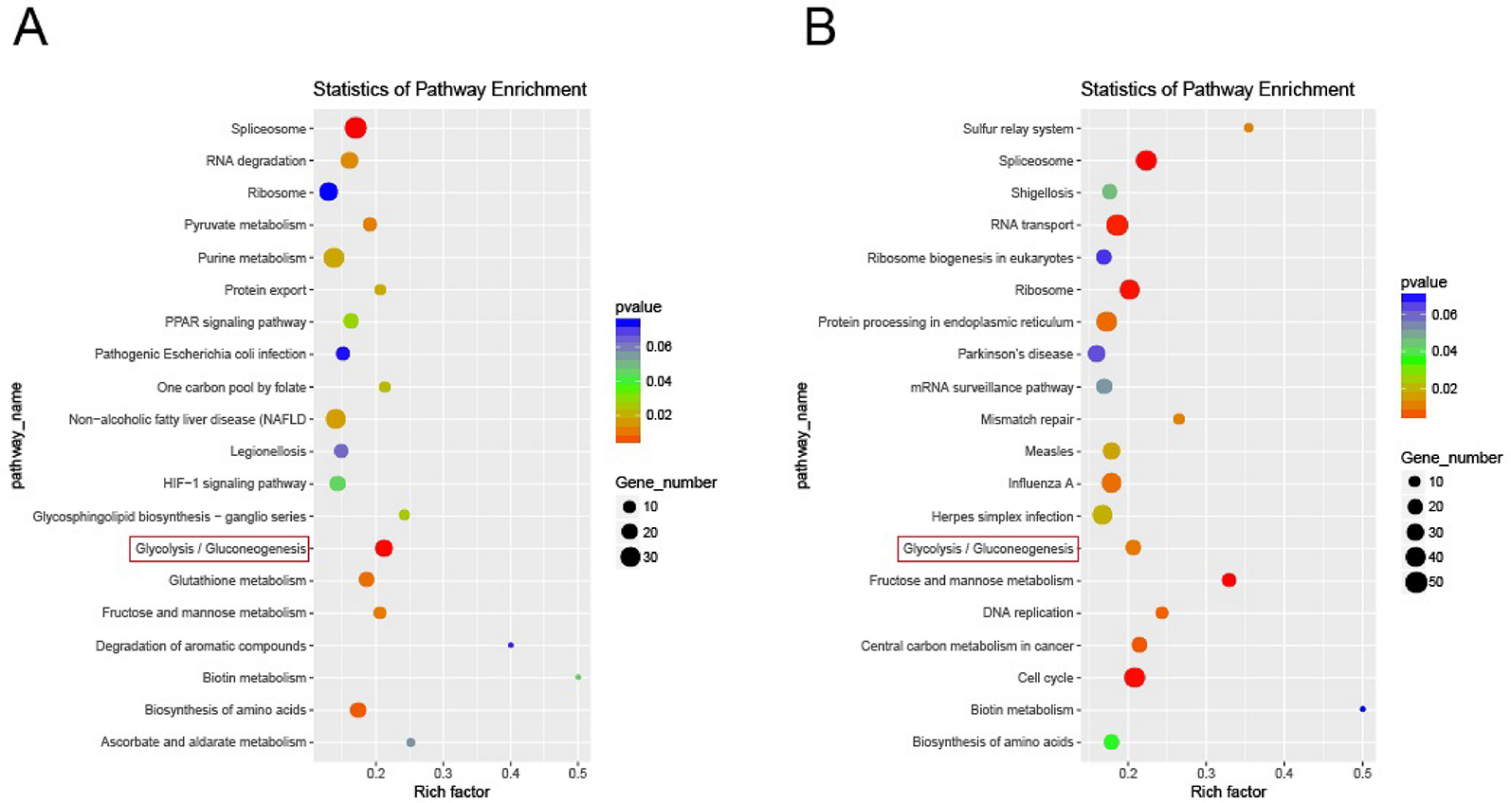
Enrichment of differentially expressed genes in KEGG pathway. Model group vs. control group **(A)**. AP-BBR group vs. model group **(B)**.

The statistical enrichment of differentially expressed genes in KEGG pathway was analyzed by local perl script and R software package ggplot2. Red indicates a significant difference, and blue indicates no significant difference. The size of the circle reflects the number of genes. Among the top 20 rich factors, pathways related to IR included the PPAR pathway, glucose metabolism pathway, and glycolysis/gluconeogenesis pathway. The results of significant differential expression analysis showed significant differences in the effect of glycolysis/GNG and other pathways on IR (**Figure 4A**). After AP-BBR intervention, there was significant difference expression in Glycolysis/GNG pathway (**Figure 4B**).

### Protein Expressions

**Figure 5:**
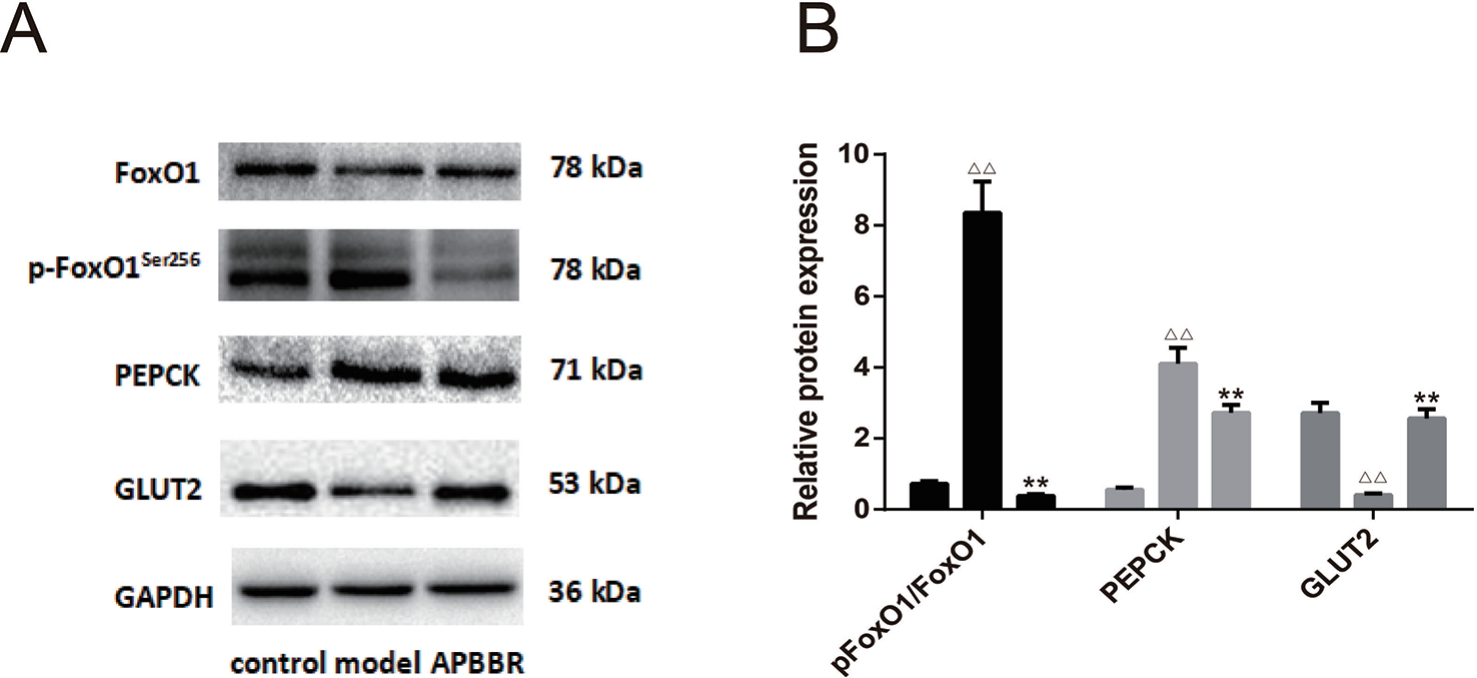
The protein expression in HepG2 cells. The protein expressions of FoxO1, p-FoxO1^Ser256^, PEPCK, and GLUT2 were detected by Western blot. The figure represents one of three experiments with similar results **(A)**. In **(B)**, ^ΔΔ^ *P* < 0.01 *vs.* control group, **P < 0.01, *vs.* model group (n = 3).

The results of Western blot in each HepG2 cell group are shown in **Figure 5**. Compared with the control group, the protein expression of p-FoxO1^Ser256^ and PEPCK in the model group was significantly increased, while the protein expression of FoxO1 and GLUT2 was significantly decreased (*n =* 3). Compared with the model group, the protein expression of p-FoxO1^Ser256^ and PEPCK in AP-BBR group was significantly decreased, while the protein expression of FoxO1 and GLUT2 was significantly increased (*n* = 3).

## Discussion

Deficiency of insulin signaling and IR in the liver has a great impact on energy balance and metabolism. Liver IR can lead to a series of metabolic abnormalities, such as hyperglycemia, dyslipidemia, and increased secretion of inflammatory factors. The inhibitory effect of insulin on hepatic glucose production is impaired during liver IR. When IR progresses to T2DM, the lack of insulin signaling molecules is common as hyperglycemia. Insulin signaling can impact T2DM by regulating glucolipid metabolism and energy homeostasis *via* action on the liver, skeletal muscle, and adipose tissue. It is reported that disruption of insulin signaling in the moue liver, skeletal muscle or adipose tissue can lead to diabetes (Boucher et al., 2014). Mice lacking insulin receptor substrate-2 (IRS-2) in the liver developed severe IR and hyperglycemia (Williams and Olefsky, 1990). All in all, according to the data of human and animal research, liver IR leads to the development of hyperglycemia and T2DM.

Astragalus membranaceus is one of the most widely used traditional Chinese herbal medicines. It can be used as an antidiabetic agent. The main component of Astragalus membranaceus is AP, which can improve overall IR (Sun et al., 2017). Coptis chinensis has been used as an anti-diabetic drug in Traditional Chinese Medicine (TCM) for centuries, and BBR is the main bioactive alkaloid of Coptis chinensis and can moderate T2DM by alleviating IR (Abd et al., 2013).

AP attenuated IR mainly by decreasing the phosphorylation of protein kinase and inhibiting GLUT4 translocation in KKAy mice (Zhang and Pugliese, 2015). AP also promoted the expression of phosphorylated PI3Kp85 and GLUT4 by inhibiting the expression of JNK and phosphorylating insulin receptor substrate-1 (Jiang et al., 2015). In addition, AP decreased serum TNF-α, MCP-1, and ICAM-1 and inhibited the expression of NF- κ B mediated inflammation gene (Gui et al., 2013).

BBR attenuated IR mainly by inhibiting the Rho GTPase signaling pathway to reduce renal inflammation in diabetic rats (Xie et al., 2013), activating various human cell lines and their promoter-dependent protein kinase C-(PKC-), and increasing insulin receptor mRNA and protein expression (Pang et al., 2015). It also inhibited liver GNG of the LKB1-AMPK-TORC2 signaling pathway and improved LPS-induced β-cell damage through LPS4-dependent JNK/NF-kB signaling pathway (Jiang et al., 2015). In addition, BBR exerted its anti-inflammatory effect through insulin sensitization, reduced cytokine production and serine phosphorylation, and increased insulin-mediated tyrosine phosphorylation (Lou et al., 2011).

The liver is the central site of glucose synthesis and metabolism, and also the main target organ for IR (Reinke and Asher, 2016). Therefore, it is very important to establish a stable and reliable IR hepatocyte model for studying the molecular mechanism of IR *in vitro* and screening drugs for prevention and treatment of IR. HepG2 cells originating from human hepatic embryonal tumor cells have similar morphological and biological functions to normal human hepatocytes, and therefore, are most commonly used for studying hepatic IR. Insulin has been widely recognized as an inducer to establish IR cell models.

In the present study, we explored the conditions in establishing the IR- HepG2 cell model and the effect of AP-BBR on IR. The glucose content in the supernatant of HepG2 cells treated with insulin for 24, 36, 48, and 72 h was measured. It was found that the optimal time for modeling was at 48 h of insulin intervention. CCK8 was used to detect the effect of AP-BBR on the viability of IR-HepG2 cells after 48 h. The results showed that AP-BBR attenuated IR in HepG2 cells.

Oxidative stress in hepatocytes refers to the pathological process of hepatocyte injury caused by excessive or/and endogenous antioxidant capacity of ·OH, H_2_O_2,_ and other active ROS, and disturbed balance between the oxidant and antioxidant systems (Cortes-Rojo and Rodriguez-Orozco, 2011). Oxidative stress causes lipid peroxidation damage, DNA oxidative damage, and abnormal protein expression in the liver by producing excessive ROS. It is involved in the pathogenesis of viral hepatitis, non-alcoholic/alcoholic hepatitis, drug-induced liver disease, and other liver diseases (Souli et al., 2015; Yu et al., 2015). When oxides or alkylates enter cells, they induce the production of a large amount of ROS. If ROS is not cleared in time, free radicals, the intermediate products of ROS, can directly act on nucleic acid, resulting in base modification and DNA strand breaks, resulting in oxidative DNA damage (Cadet et al., 2011; Rinna et al., 2015). Alterations in ROS and apoptosis of HepG2 cells were detected by flow cytometry. The results showed that the level of ROS increased significantly in IR-HepG2 cells and decreased after AP-BBR treatment. However, AP-BBR treatment had no significant effect on apoptosis of HepG2 cells.

Ca^2+^ is an important second messenger in cells. There is a close interaction between the ROS signaling system and the calcium signaling system in the intracellular environment. On the one hand, ROS can change the amplitude and temporal and spatial characteristics of local and whole-cell calcium signals by modifying the key protein components of the calcium signaling system, such as voltage-dependent calcium and intracellular calcium release channels. On the other hand, Ca^2+^ can accelerate mitochondrial metabolism by regulating the activities of dehydrogenase in tricarboxylic acid cycle (Rizzuto and Pozzan, 2006), ATP synthase, and adenine nucleoside transporter so as to accelerate mitochondrial metabolism and increase ROS production. [Ca^2+^]i can adjust mitochondrial proton driving force (Δ ψ m and Δ PH) and affect mitochondrial energy conversion and ROS production. Ca^2+^ is also involved in the regulation of cell cycle, cell differentiation, muscle contraction, cell movement, exocytosis, endocytosis, chemical chemotaxis, synaptic transmission and synaptic plasticity, and apoptosis (Liu and O'Rourke, 2009). [Ca^2+^]i is both a survival signal and a death signal. [Ca^2+^]i in cells is harmful to cells when it is too high or too low, so cell [Ca^2+^]i is under very strict regulation and control. The dynamic change of cell [Ca^2+^]i is the core link in cell signal transduction and regulatory network (Feske et al., 2001). Physiological levels of ROS and Ca^2+^ to adapt to the changes in energy requirements continue to adjust adaptively, so as to regulate the dynamic balance of mitochondrial morphology and distribution.

When the regulation of Ca^2+^ and ROS at physiological level fails to adapt to the change of energy demand, the abnormal level of Ca^2+^ and ROS leads to mitochondrial fusion and division, thus disrupting the balance of dynamic distribution, causing dysfunction of mitochondrial oxidative phosphorylation function and further affecting the cell function. The body is in a pathological state, and at the same time, mitochondrial oxidative phosphorylation dysfunction further leads to abnormal levels of Ca^2+^ and ROS, forming a vicious circle. ROS plays an important role in regulating cell proliferation, differentiation, apoptosis and cell senescence. Low concentrations of H_2_O_2_ can be used as intracellular signaling molecules to initiate gene transcription, thus inducing cell growth. High concentrations of ROS oxidized lipid, protein, and DNA can cause damage to cell integrity (Scherz-Shouval and Elazar, 2007).

Apoptosis induced by H_2_O_2_ is closely related to calcium overload. The molecular mechanism of apoptosis induced by oxidative stress is very complex and involves many signal transduction pathways, including the classical mitochondrial pathway (Chiara et al., 2012), death receptor pathway (Gomez-Quiroz et al., 2008) and endoplasmic reticulum pathway (Ip et al., 2011). Oxidative stress damage of cells leads to the release of a large amount of Ca^2+^ from the endoplasmic reticulum accompanied by endoplasmic reticulum stress, leading to unfolded protein reaction (UPR), which is used to reconstruct the normal function of endoplasmic reticulum (Greotti et al., 2019). If the intracellular Ca^2+^ concentration continues to increase and endoplasmic reticulum stress lasts for a prolonged time or severely, Ca^2+^-dependent kinases and phosphatase, such as calpain, caspase-12, and caspase-3 cascade reactions, will be activated, eventually leading to apoptosis (Das et al., 2006). In our experiment, change in the intracellular H_2_O_2_ concentration in IR-HepG2 cells was detected after AP-BBR treatment. The results showed that the H_2_O_2_ content of IR-HepG2 cells increased after modeling and decreased after AP-BBR treatment. The change in intracellular calcium flow in IR-HepG2 cells was rapid and reduced after AP-BBR treatment. These results suggest that Ca^2+^ was activated in the IR HepG2 cell model, and AP-BBR could inhibit the activation of Ca^2+^ to a certain extent.

The IR-INS-1 cell model in our previous study (Zhu-jun et al., 2017) showed that combined use of AP and BBR could promote basic secretion of IR-INS-1 cells significantly and showed a better therapeutic effect than that when either of them was used alone. The results showed that the compatibility of the two drugs had an optimal synergistic effect, and that AP-BBR could promote the proliferation of IR-HepG2 cells and reduce intracellular calcium flow, though the underlying mechanism remains unclear at present.

Insulin is a key hormone that suppresses GNG. It plays a vital role in the regulation of hepatic glucose output. Insulin regulates hepatic GNG by transcriptional modulation and activation of the relative signaling pathways (Hatting et al., 2018). Even an indirect effect of insulin on extra hepatic tissues can be sufficient to maintain normal glucose metabolism (O-Sullivan et al., 2015). T2DM is characterized by excess glucagon and IR. In T2DM patients, insulin could not suppress glucagon secretion or hepatic glucose production, which underscores the importance of determining the GNG rate in the potential treatment for T2DM.

Forkhead box protein O1 (FoxO1) is a member of the forehead box transcription factor class O (FOXO) family, and a direct transcriptional regulator of GNG. It is regulated by insulin reciprocally and plays a major role in regulating insulin sensitivity (Tikhanovich et al., 2013). The activity of FOXO1 as a regulator of blood glucose is modulated by several mechanisms including phosphorylation, acetylation, and deacetylation balance, as well as some other more novel mechanisms (Zhou et al., 2011).

In the high blood glucose state, insulin is released from the pancreas into the blood stream. Insulin signaling causes the activation of PI3kinase and the subsequent production of PIP3 phosphorylates Akt. Akt then phosphorylates FoxO1 to its nuclear exclusion, and is ubiquitinated and degraded by the proteosome (Matsuzaki et al., 2003). That is to say, FOXO1 is a counter of insulin-induced Akt activation in glycogen synthesis-GNG balance, which can subsequently suppress GNG during the fed state (Daitoku and Fukamizu, 2007).

Phosphoenolpyruvate carboxykinase (PEPCK) is an enzyme in the lyase family used in the metabolic pathway of GNG. It converts oxaloacetate into phosphoenolpyruvate and carbon dioxide (Mendez-Lucas et al., 2014).

The transcriptional induction of gluconeogenic enzyme PEPCK-C is an irreversible step of GNG, which therefore is of great importance in glucose homeostasis, as evidenced by the overexpression of PEPCK-C in T2DM laboratory mice. The role that PEPCK-C plays in GNG may be mediated by the citric acid cycle, the activity of which was found to be directly related to PEPCK-C abundance. Previous studies suggested that PEPCK-C level alone was not highly correlated with GNG in the mouse liver (Marandel et al., 2019). While the mouse liver almost exclusively expresses PEPCK-C, humans equally present a mitochondrial isozyme (PEPCK-M). Therefore, the role of PEPCK-C and PEPCK-M in GNG may be more complex and involve more factors than was previously believed.

Glucose metabolism depends on the uptake of glucose by cells. However, glucose is not free to enter cells through the lipid bilayer structure of the cell membrane. Glucose uptake by cells requires the help of glucose transporter (glucose transporters) on the cell membrane, which is known as the glucose transporter (GLUT) transport function.

GLUTs facilitate the movement of glucose from the plasma membrane across cell membranes. Member 2 (SLC2A2) of Glucose transporter 2 (GLUT2) is a principal transmembrane transporter for transfer of glucose between the liver and blood (Gould et al., 1991). It is of great significance for hepatic glucose uptake. Unlike GLUT4, GLUT2 does not rely on insulin for facilitation of glucose diffusion.

GLUT2 has low affinity (high Km, ca. 15-20 mM) for glucose, but it has high capacity for glucose. What's more, GLUT2 isoform is found abundant in the liver and pancreatic β-cells, so it has been suggested to function as a glucose sensor (Jung et al., 2006). GLUT2 may play a minor role in human β-cells, and can be an efficient glucose carrier so as to improve glucose and insulin metabolism.

In our study, the protein expression of p-FoxO1^Ser256^ and PEPCK in IR-HepG2 cells was increased, but the protein expression of FoxO1 and GLUT2 was decreased, and AP-BBR could reverse the above protein expression changes. The results suggest that AP-BBR attenuates IR in IR-HepG2 cells probably *via* the regulation of the GNG signaling Pathway.

## Data Availability Statement

The datasets generated for this study can be found in the NCBI GEO https://www.ncbi.nlm.nih.gov/geo/query/acc.cgi?acc=GSE139929.

## Author Contributions

Z-JM generated the data, wrote the manuscript, and approved its final version. Z-JM and L-PQ designed the study. Z-JM, ML, and XZ carried out the experiments. All authors have read and approved the final manuscript.

## Funding

This study was supported by a grant from the National Natural Science Foundation of China (No. 81603351).

## Conflict of Interest

The authors declare that the research was conducted in the absence of any commercial or financial relationships that could be construed as a potential conflict of interest.

## Abbreviations

IR, Insulin resistance; T2DM, type 2 diabetes mellitus; GNG, Gluconeogenesis; IR-HepG2, Insulin resistance HepG2 cells; AP, Astragalus polysaccharide; BBR, Berberine; AP-BBR, Astragalus polysaccharide plus berberine; ROS, reactive oxygen species; FoxO1, Forkhead box protein O1; PEPCK, Phosphoenolpyruvate carboxykinase; GLUT2, Glucose transporte; 2PPAR, Peroxisome proliferators-activated receptor; IRS-2, Insulin receptor substrate-2; GLUT4, Glucose transporter 4; UPR, Unfolded protein reaction.

## Supplementary Material

Supplementary Table 1 The raw data of intracellular Ca^2+^ fluorescence intensity.
